# Interfacial coupling effect of Cr_2_O_3_ on the magnetic properties of Fe_72_Ga_28_ thin films

**DOI:** 10.1038/s41598-021-92640-y

**Published:** 2021-06-28

**Authors:** I. Hontecillas, M. Maicas, J. P. Andrés, R. Ranchal

**Affiliations:** 1 Dpto. Física de Materiales, Fac. CC. Físicas, Universidad Complutense de Madrid, Ciudad Universitaria s/n, 28040 Madrid, Spain; 2 Institute for Optoelectronic Systems and Microtechnology (ISOM), Polytechnic University of Madrid (UPM), Avenida Complutense 30, 28040 Madrid, Spain; 3 Dept Appl Phys, Univ Castilla La Mancha, Inst Reg Invest Cient Aplicada IRICA, 13071 Ciudad Real, Spain; 4 Instituto de Magnetismo Aplicado, UCM-ADIF-CSIC, 28232 Las Rozas, Spain

**Keywords:** Materials science, Nanoscience and technology, Physics

## Abstract

Here it is investigated the effect of the antiferromagnet Cr_2_O_3_ on the magnetic properties of ferromagnetic Fe_72_Ga_28_ thin films. Sputtered Fe_72_Ga_28_ layers have their magnetization in the sample plane with a magnetic fluctuation that gives rise to magnetic ripple. In order to turn its magnetization into the out of plane (OOP) direction, it has been magnetically coupled with Cr_2_O_3_ that has magnetic moments along the *c*-axis, that is the perpendicular direction when properly aligned. Cr_2_O_3_ has been obtained from Cr oxidation, whereas Fe_72_Ga_28_ has been deposited on top of it by sputtering in the ballistic regime. Although a uniaxial in-plane magnetic anisotropy is expected for Fe_72_Ga_28_ thickness above 100 nm, the interfacial coupling with Cr_2_O_3_ prevents this anisotropy. The formation of stripe domains in Fe_72_Ga_28_ above a critical thickness reveals the enhancement of the out of plane component of the Fe_72_Ga_28_ magnetization with respect to uncoupled layers. Due to the interface coupling, the Fe_72_Ga_28_ magnetization turns into the out-of-plane direction as its thickness is gradually reduced, and a perpendicular magnetic anisotropy of 3·10^6^ erg·cm^−3^ is inferred from experimental results. Eventually, the coupling between Cr_2_O_3_ and Fe_72_Ga_28_ promotes an exchange-bias effect that has been well fitted by means of the random field model.

## Introduction

Control of the magnetization is an important issue for the development of pioneering magnetic devices. Typically, thin films have the magnetization in the sample plane in order to reduce the energy of the system^1^. However, in many applications as for example: high density magnetic storage, spintronic devices, non-volatile random access memories, logic devices, skyrmions or sensors, materials with OOP magnetization are desirable^2–6^.

Systems with large perpendicular magnetic anisotropy (PMA) such as L1_0_ FePt and CoPt thin films are extensively investigated but, their large coercivity and switching fields can represent a drawback for their integration in devices^7,8^. Therefore, it is of interest the investigation of other materials with PMA, or the possibility of turning the magnetization into the OOP direction. Stripe domains appear above a critical thickness when a moderate PMA is present^9^. In permalloy films, stripes have been observed because of columnar growth^10^, but they have also been promoted when coupled with NdCo^11^. Recently, the magnetization direction has been tuned in Fe–N layers by ion implantation and heat treatment conditions^9^, and by annealing in Fe_87_Si_9_B_13_^12^. Even more notably, the control of the magnetic anisotropy by means of voltage has been observed at magnetic transition metal/oxide interfaces^2^.

FeGa alloys are extensively studied because of their large magnetostriction constant and low coercivity^13–16^. Also interesting is the possibility in sputtered Fe_72_Ga_28_ layers of controlling the in-plane magnetic anisotropy by growth conditions^17–19^ or by thermal treatments combined with mechanical stress^20^. Molecular beam epitaxy (MBE) FeGa can exhibit domain stripes due to a low PMA^21–23^, but the general behavior observed by magnetic force microscopy (MFM) in FeGa deposited either by electrodeposition^24^, sputtering^25^ or even MBE^26^ is a magnetic ripple due to magnetic fluctuations in layers with magnetization in the sample plane.

In this work, we have explored the possibility of turning the Fe_72_Ga_28_ magnetization into the OOP direction when appropriately coupled with Cr_2_O_3_, an antiferromagnet that has its magnetic moments along the hexagonal *c*-axis being possible to have moments in the perpendicular direction when properly aligned^27–29^. Although Cr_2_O_3_ has already been coupled with ferromagnetic metals^30–32^, the interaction with Fe_72_Ga_28_ seems not a trivial problem. Fe_72_Ga_28_ has already been coupled with TbFe_2_ in [Fe_72_Ga_28_/TbFe_2_] multilayers to turn its magnetization into the OOP direction^33,34^. However, only a tilt of the magnetization of around 25° with respect to the sample plane was achieved despite the large PMA of TbFe_2_^33,34^. Here we show that by means of a suitable experiment design in terms of layer thickness and materials, it is possible to reach an effective interfacial interaction that enables to turn the Fe_72_Ga_28_ magnetization into the OOP direction. In addition, because of the interfacial interaction we have observed exchange-bias effect in the perpendicular direction being possible to fit the experimental exchange-bias fields (*H*_*E*_) by means of the random field model^35–37^.

## Experimental section

30 nm-thick Cr_2_O_3_ was synthetized from the evaporation of Cr on glass substrates that was subsequently oxidized in oxygen atmosphere at 750 °C during 3 h. Fe_72_Ga_28_ layers with a thickness ranging from 240 to 20 nm were grown by the DC magnetron sputtering technique in the ballistic regime at room temperature on top of the Cr_2_O_3_. The sputtering deposition was carried out in oblique incidence with an angle between the vapor beam and the perpendicular to the sample of about 25° and a distance of 9 cm between target and substrate. This direction of the vapor beam within the sample plane is taken as the reference direction to control the direction of the in-plane uniaxial anisotropy axis when created^16,17,38^. Fe_72_Ga_28_ films were deposited from a target with a composition of Fe_72_Ga_28_ with a diameter of 5 cm and a thickness of 2 mm using an Ar pressure of 3·10^−3^ mbar and a growth power of 90 W in all cases. A 20-nm thick Mo layer was used as a capping layer to avoid FeGa oxidation. Mo was also deposited with a power of 90 W and with an Ar pressure of 3·10^−3^ mbar. Therefore, the structure of the studied samples is: glass/ Cr_2_O_3_/Fe_72_Ga_28_/Mo. For further comparisons, we have also deposited single Fe_72_Ga_28_ layers with thickness of 20 and 150 nm between Mo buffer and capping layers to avoid oxidation.

We have used X-ray diffractometry (XRD) in the Bragg–Brentano configuration to study the structural properties. Measurements were performed in a Philips X’Pert MPD using the Cu K_α_ wavelength (1.54056 Å). A Digital Instruments Nanoscope IIIa instrument was used to obtain MFM images. We monitored the cantilever’s phase of oscillation while the magnetic tip was scanning the sample surface to work in the phase detection mode. The distance between sample and surface was 40 nm on average (lift mode)^33^. The MFM measurements were performed without magnetic field after an OOP magnetic field of 10 kOe was applied. The topography of the samples has also been obtained in this microscope working as an Atomic Force Microscope (AFM).

In-plane and OOP hysteresis loops were performed in a vibrating sample magnetometer (VSM) from LakeShore 7304 at room temperature. In the sample plane, we measured loops at different angles between the applied magnetic field and the in-plane reference direction. As a reference direction, we considered as 0° the beam incidence direction in the sample plane^17,38^. Hysteresis loops at 5 K were measured in a SQUID EverCool MPMS SQUID magnetometer from Quantum Design after field-cooling (FC) at 1 kOe from 360 K and zero-field-cooling (ZFC) at 0 kOe from 360 K. To reduce any hypothetical systematic error from the SQUID, a field step of 10 Oe has been used in the low field region of the hysteresis loops. Also, the comparison between FC and ZFC hysteresis loops has been used to cross-check the shift in the field axis. At low temperature, only OOP loops were recorded. Magnetic moment versus temperature was also measured with the magnetic field in the perpendicular direction.

## Results and discussion

**Figure 1:**
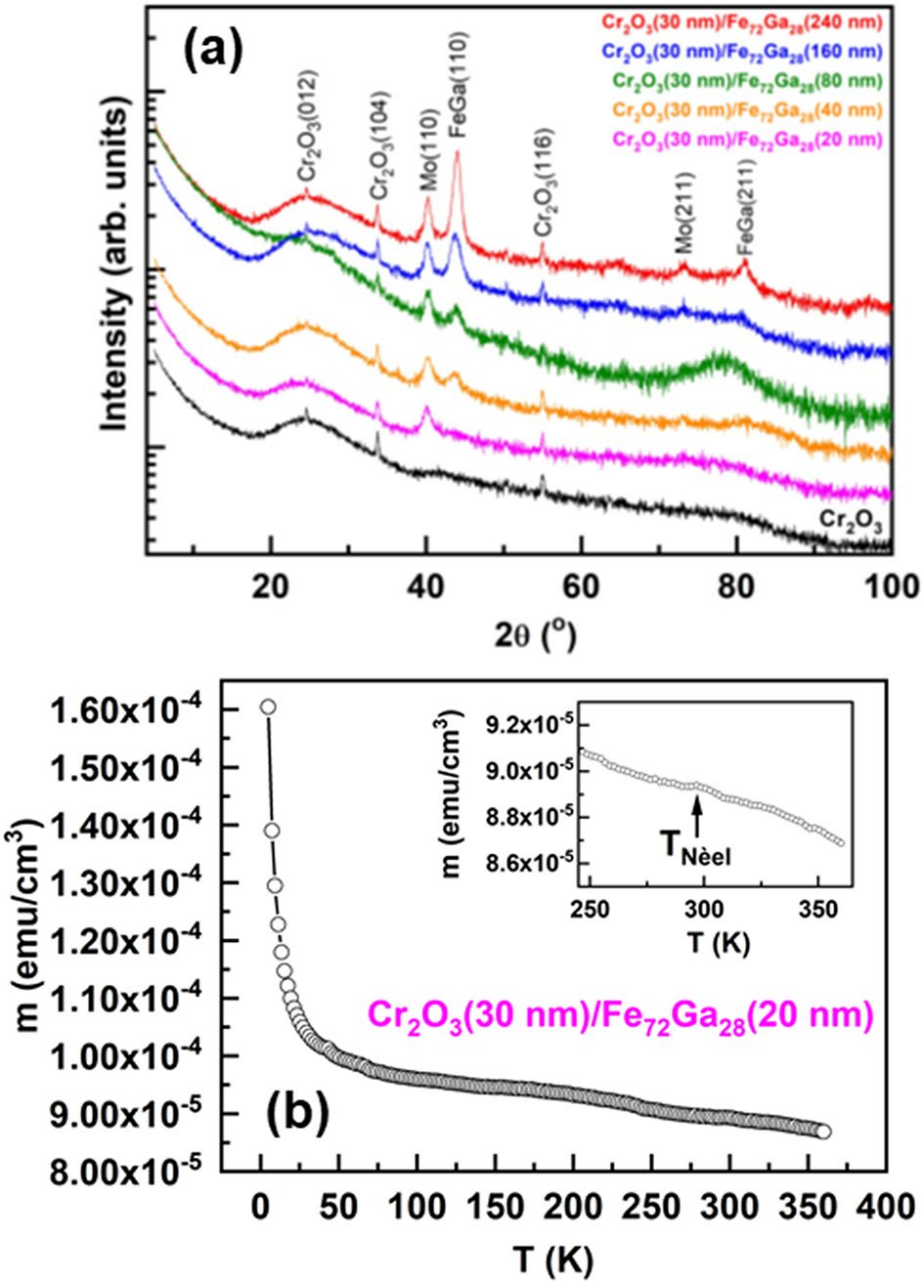
(**a**) XRD diffraction patterns of the samples studied in this work. The measurement for a 30 nm-thick Cr_2_O_3_ layer on glass has been included for further comparisons. Curves are shifted for clarity. (**b**) Magnetic moment versus temperature for the Cr_2_O_3_/Fe_72_Ga_28_(20 nm) sample. Inset: Detail of the measurement.

In the XRD measurements we have only observed diffractions peaks related to either Cr_2_O_3_, FeGa or Mo (Fig. 1a). We have analyzed the possibility of Fe_72_Ga_28_ oxidation because of its growth on top of Cr_2_O_3_, but XRD measurements do not show evidences of oxidation within the resolution technique. The diffraction peaks related to FeGa are similar to what we have previously reported about sputtered Fe_72_Ga_28_ thin films being the (110) the main diffraction peak^16–19^. For Fe_72_Ga_28_ we can determine the lattice parameter (*a*) thanks to Bragg’s law:

1 $$n = 2d_{{hkl}} sin\theta$$

where ${d}_{hkl}$ is the family of planes,$\theta$ the diffraction angle, and $\lambda$ the radiation wavelength (Cu K_α_ in this case). In the cubic system, we can obtain *a* from *d*_*hkl*_ since:

2 $$a = d_{{hkl}} \left( {h^{2} + k^{2} + l^{2} } \right)$$

where *h*, *k*, and *l* are the Miller indexes of the family of planes. From experimental results for the (110) peak we have determined a lattice parameter of 2.90 Å, in agreement with previous works^17,18^.

**Figure 2:**
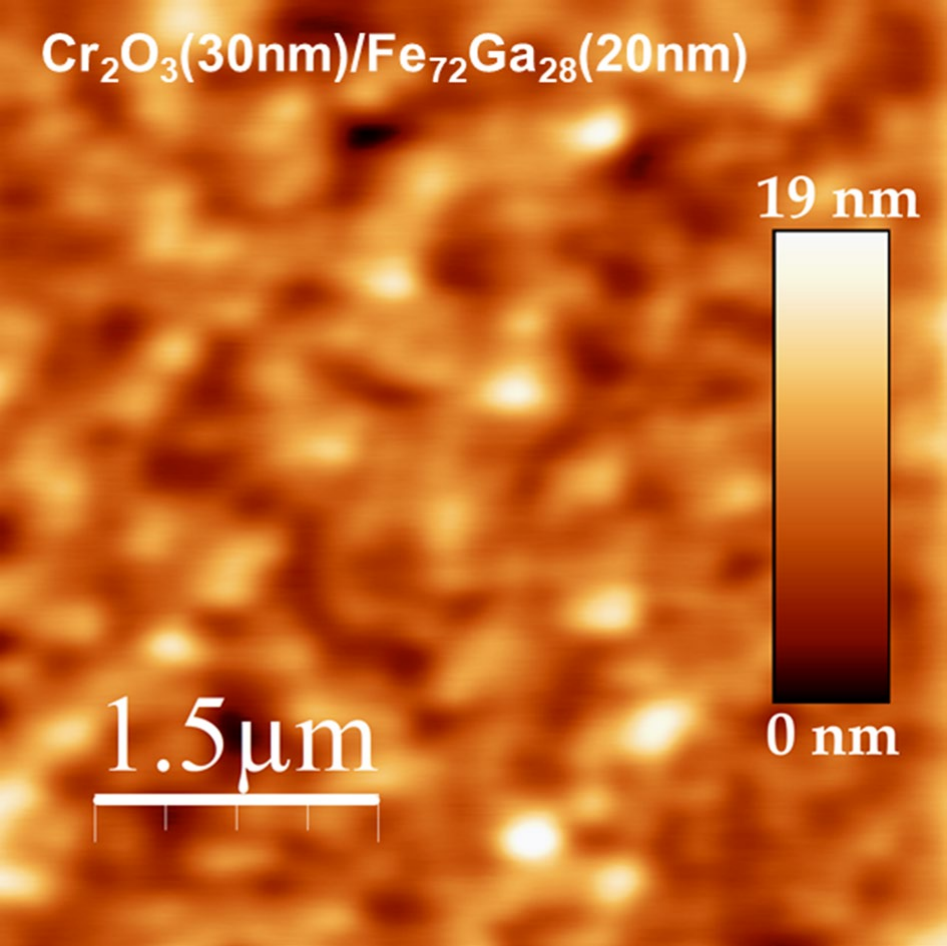
Morphology of the sample Cr_2_O_3_(30 nm)/Fe_72_Ga_28_(20 nm) measured by AFM in an area of 5 μm × 5 μm.

On the other hand, the diffraction peaks of Cr_2_O_3_ show no variations upon the deposition of Fe_72_Ga_28_ on top of it as it can be inferred from the comparison with a single layer of Cr_2_O_3_ deposited in the same growth conditions (Fig. 1a). Experimental diffraction peaks for Cr_2_O_3_ appear at the same diffraction angles than for the file used for identification (01-084-0312) and therefore, it has a rhombohedral structure with lattice parameters *a* = *b* = 4.95 Å and *c* = 13.56 Å. Cr_2_O_3_ layers are not fully *c*-oriented since (012), (104) and (116) diffraction peaks for Cr_2_O_3_ have been detected by XRD. However, it is expected a magnetic contribution along the perpendicular direction due to these family of planes. The Nèel temperature (*T*_*N*_) of the Cr_2_O_3_ has been obtained from the measurement of the magnetization as a function temperature (Fig. 1b). The experimental value of *T*_*N*_ = 297 K is just 10 K below that of bulk Cr_2_O_3_, *T*_*N*_ = 307 K. Finally, we have also analysed the morphology of the samples by AFM (Fig. 2) being obtained a root mean square (rms) roughness of around 2 nm for the final stack.

**Figure 3:**
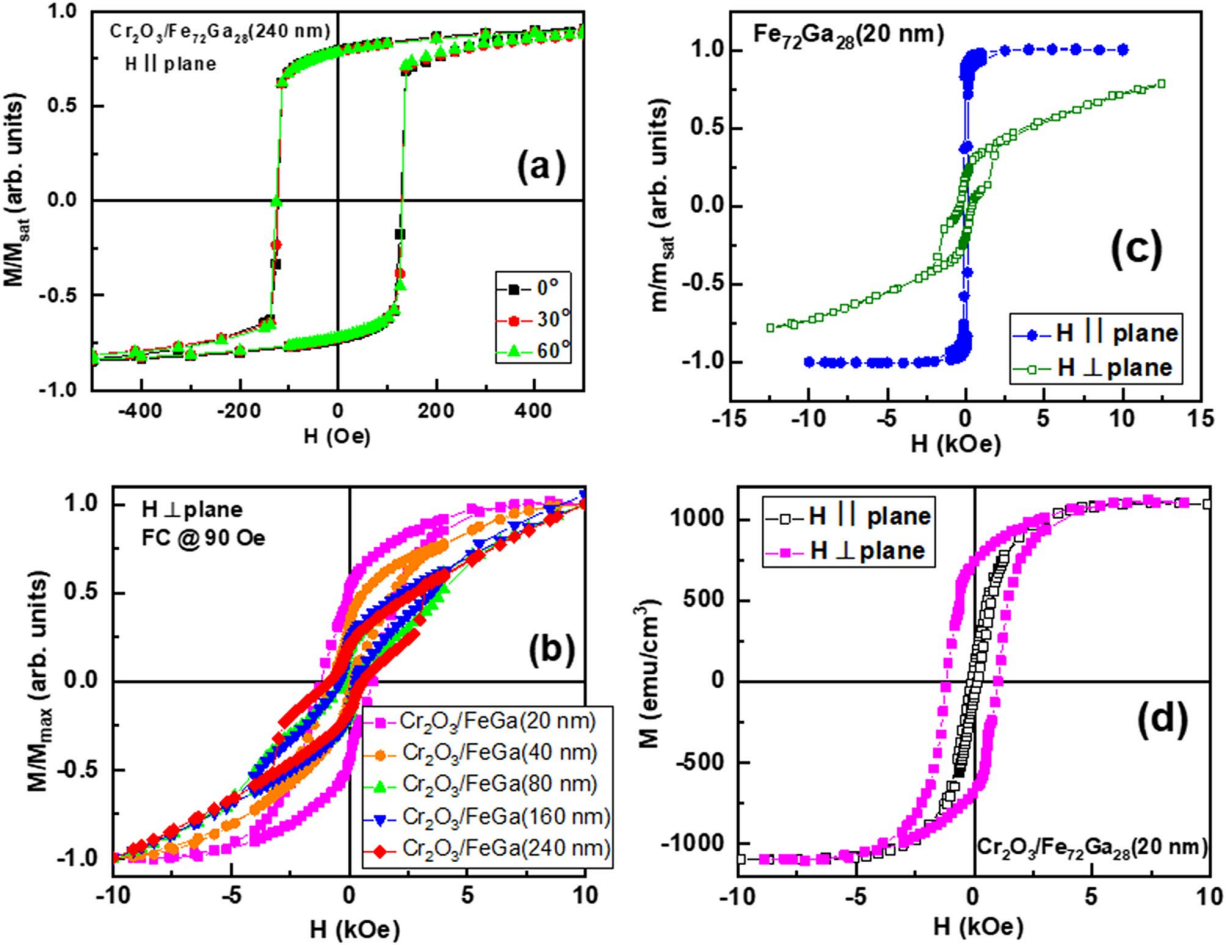
(**a**) In-plane room temperature hysteresis loops recorded for different angles between the reference direction taken as the reference beam direction and the applied magnetic field (filled square) 0°, (filled circle) 30°, and (filled triangle) 60° for the Cr_2_O_3_/Fe_72_Ga_28_(240 nm) sample. (**b**) OOP hysteresis loops recorded at room temperature for samples with different FeGa thickness (filled square) 20 nm, (filled circle) 40 nm, (filled triangle) 80 nm, (filled down triangle) 160 nm, and (filled diamond) 240 nm. (**c**) In plane and perpendicular hysteresis loops at room temperature for an uncoupled 20 nm Fe_72_Ga_28_ thin film. (**d**) Comparison of in plane (open square) and perpendicular (filled square) hysteresis loops at room temperature for the Cr_2_O_3_/Fe_72_Ga_28_(20 nm) sample.

Sputtered Fe_72_Ga_28_ layers deposited in the ballistic regime develop in-plane magnetic anisotropy above 100 nm^16,18^. Nevertheless, the coupling with Cr_2_O_3_ completely eliminate this in-plane anisotropy even for thicknesses well above 100 nm (Fig. 3a). This can be understood considering that a sample will show PMA if two conditions are fulfilled: i) it is magnetically isotropic in the sample plane, and ii) the OOP direction is an easy axis in comparison to any direction in the sample plane. Therefore, the absence of in-plane magnetic anisotropy is a necessary condition for the PMA to be developed. We have ruled out that magnetostriction and strain have any effect on this modification of the magnetic anisotropy since we have not observed differences of the Fe_72_Ga_28_ lattice parameter with respect to uncoupled single layers. In fact, when strain has been used to manipulate the magnetic anisotropy in Ga-rich FeGa thin films, only the direction within the sample plane was modified^20^.

The VSM hysteresis loops reveal the change of the magnetization direction from in-plane to OOP as the Fe_72_Ga_28_ thickness is reduced (Fig. 3b). We can quantitively monitor this evolution by means of the OOP squareness (*M*_*r*_/*M*_*max*_) defined as the ratio between the remanence (*M*_*r*_) and the maximum magnetization (*M*_*max*_) obtained in the perpendicular hysteresis loops. In Table 1 we can observe how the squareness increases as the Fe_72_Ga_28_ thickness is reduced, mostly for a thickness below 80 nm. The OOP squareness and hysteresis loops of coupled layers can be compared with those of a Fe_72_Ga_28_ thin film grown in similar conditions (Table 1; Fig. 3c). The OOP squareness of a single layer is much lower with respect to that of a Fe_72_Ga_28_ with the same thickness but coupled to Cr_2_O_3_. Finally, in Fig. 3d it is presented the comparison between in-plane and OOP hysteresis loops for the Cr_2_O_3_/Fe_72_Ga_28_(20 nm) sample to show the magnetization direction has been turned into the perpendicular direction becoming the OOP direction an easy axis.

**Table 1:** OOP squareness (*M*_*r*_/*M*_*max*_) calculated from the perpendicular hysteresis loops as a function of the Fe_72_Ga_28_ thickness in the Cr_2_O_3_/Fe_72_Ga_28_ system, and for an uncoupled single Fe_72_Ga_28_ layer. Perpendicular magnetic anisotropy (K_FM_) for the Cr_2_O_3_/Fe_72_Ga_28_ system in those samples in which the OOP direction is a clear easy axis.

Fe_72_Ga_28_ thickness (nm)	Cr_2_O_3_/Fe_72_Ga_28_ system FeGa thickness (nm)					Single Fe_72_Ga_28_ thickness (nm)
	20	40	80	160	240	20
*M*_*r*_/*M*_*max*_ (OOP direction)	0.5	0.3	0.2	0.2	0.2	0.2
Perpendicular *K*_*FM*_ (erg/cm^3^)	2.8·10^6^	3.3·10^6^	–	–	–	–

The perpendicular magnetic anisotropy, *K*_*FM*_, can be experimentally inferred taking into account that:

3 $$K_{{FM}} = \frac{{M_{{FM}} \cdot H_{K} }}{2}$$

being *M*_*FM*_ the saturation magnetization of the Fe_72_Ga_28_, and *H*_*K*_ the anisotropy field in the hard direction. For the samples with Fe_72_Ga_28_ thickness of 20 nm and 40 nm the in-plane is a hard axis, and we have obtained an average value of *K*_*FM*_ of 3·10^6^ erg cm^−3^ (Table 1) for a *M*_*FM*_ = 1100 emu cm^−3^^26^. For higher Fe_72_Ga_28_ thickness, the OOP direction is not a clear easy axis due to the progressive turning of the magnetization into the perpendicular direction as the FeGa thickness is reduced.

**Figure 4:**
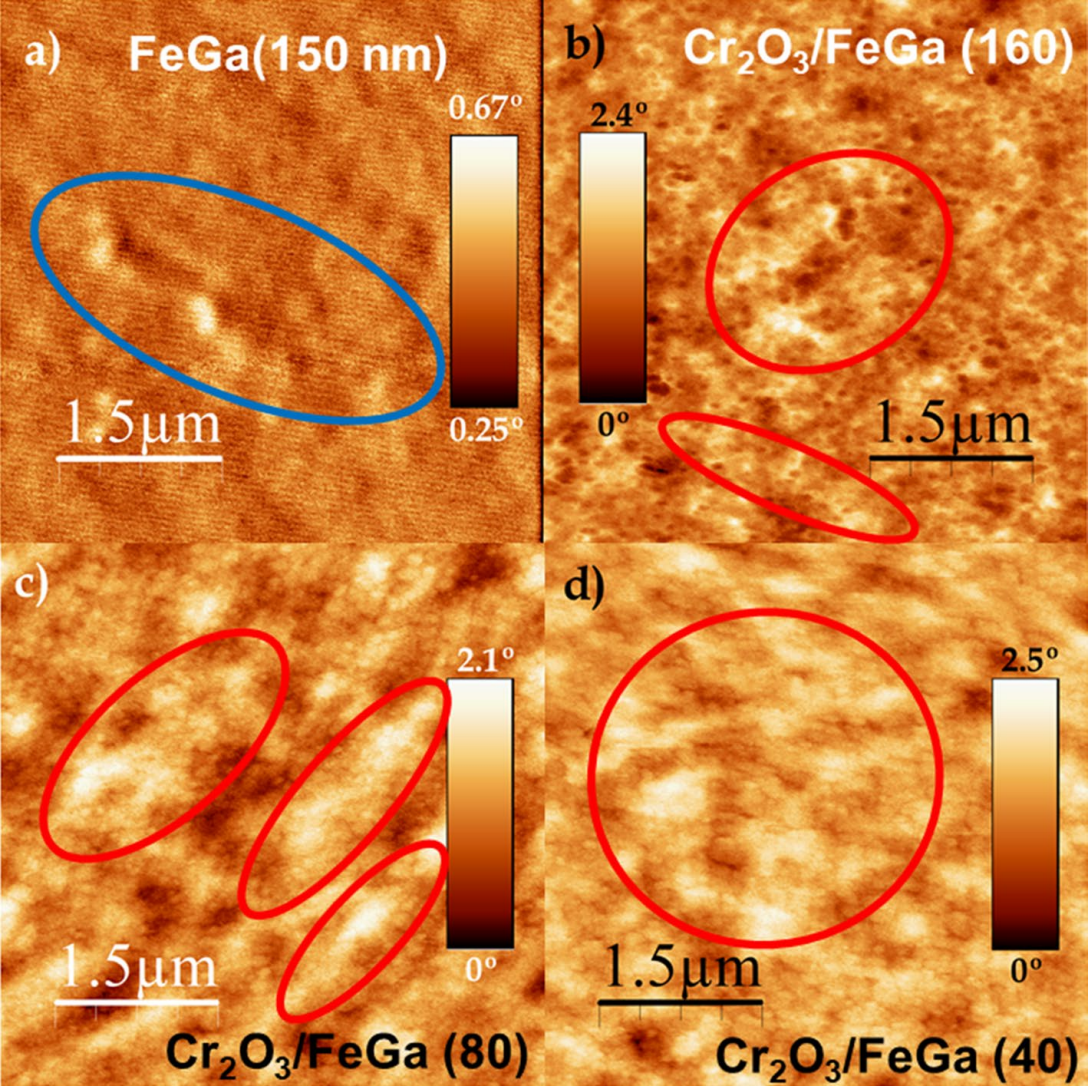
MFM images taken at remanence after an applied magnetic field of 10 kOe was applied in the perpendicular direction. (**a**) 150 nm-thick uncoupled Fe_72_Ga_28_ layer. With a blue line it is indicated an example of the magnetic contrast known as ripple. (**b**) Cr_2_O_3_/Fe_72_Ga_28_(160 nm), (**c**) Cr_2_O_3_/Fe_72_Ga_28_(80 nm), and (**d**) Cr_2_O_3_/Fe_72_Ga_28_(40 nm). In (**b**), (**c**) and (**d**), a red line is used to highlight examples of areas where magnetic stripes can be observed.

MFM images can also be used to monitor the influence of the Cr_2_O_3_ on the Fe_72_Ga_28_ magnetic behavior (Fig. 4). When Fe_72_Ga_28_ is no coupled (Fig. 4a), it is observed the magnetic contrast known as magnetic ripple in agreement with previous works^24–26^. However, due to the interfacial coupling with Cr_2_O_3_, the OOP component of the magnetization of Fe_72_Ga_28_ is enhanced, and stripe domains start to be visible in the MFM images (Fig. 4b–d).

In ferromagnetic materials with PMA, the quality factor *Q* is defined as^39^:

4 $$Q = K_{{FM}} /2\pi M_{{FM}}^{2}$$

For materials with moderate or low PMA, *Q* < 1, stripe domains appear above a critical thickness (*t*_*cr*_):

5 $$t_{{cr}} = 2\pi \sqrt {A_{{ex}} /K_{{FM}} }$$

where *A*_*ex*_ is the exchange energy per unit length. When *Q* > 0.1, stripe domains are wider than the layer thickness, whereas they exhibit a periodicity equals to the layer thickness if *Q* < 0.1.

Taking into account the experimental value inferred for the perpendicular magnetic anisotropy, *K*_*FM*_ = 3·10^6^ erg/cm^3^, and *M*_*FM*_ = 1100 emu cm^−3^^26^, it is inferred a *Q* of 0.3 in our samples and therefore, stripes are expected above a critical thickness. Considering *A*_*ex*_ = 1.7·10^−6^ erg cm^−1^ from the literature^22,23,40^, and using Eq. (5), it is obtained an experimental *t*_*cr*_ of 44 nm. This is in agreement with our experimental findings in which stripes have only been observed for Fe_72_Ga_28_ thickness ≥ 40 nm. In fact, if we take this experimental value as *t*_*cr*_, a *K*_*FM*_ of 4·10^6^ erg cm^−3^ is calculated, close to the experimental inferred value from experimental hysteresis loops, 3·10^6^ erg cm^−3^. Finally, from the MFM images we have obtained a stripe period between 110 and 125 nm by means of the power spectral density (supplementary information). This stripe periodicity higher than the layer thickness is consistent with the quality factor *Q* higher than 0.1 calculated in our samples.

**Figure 5:**
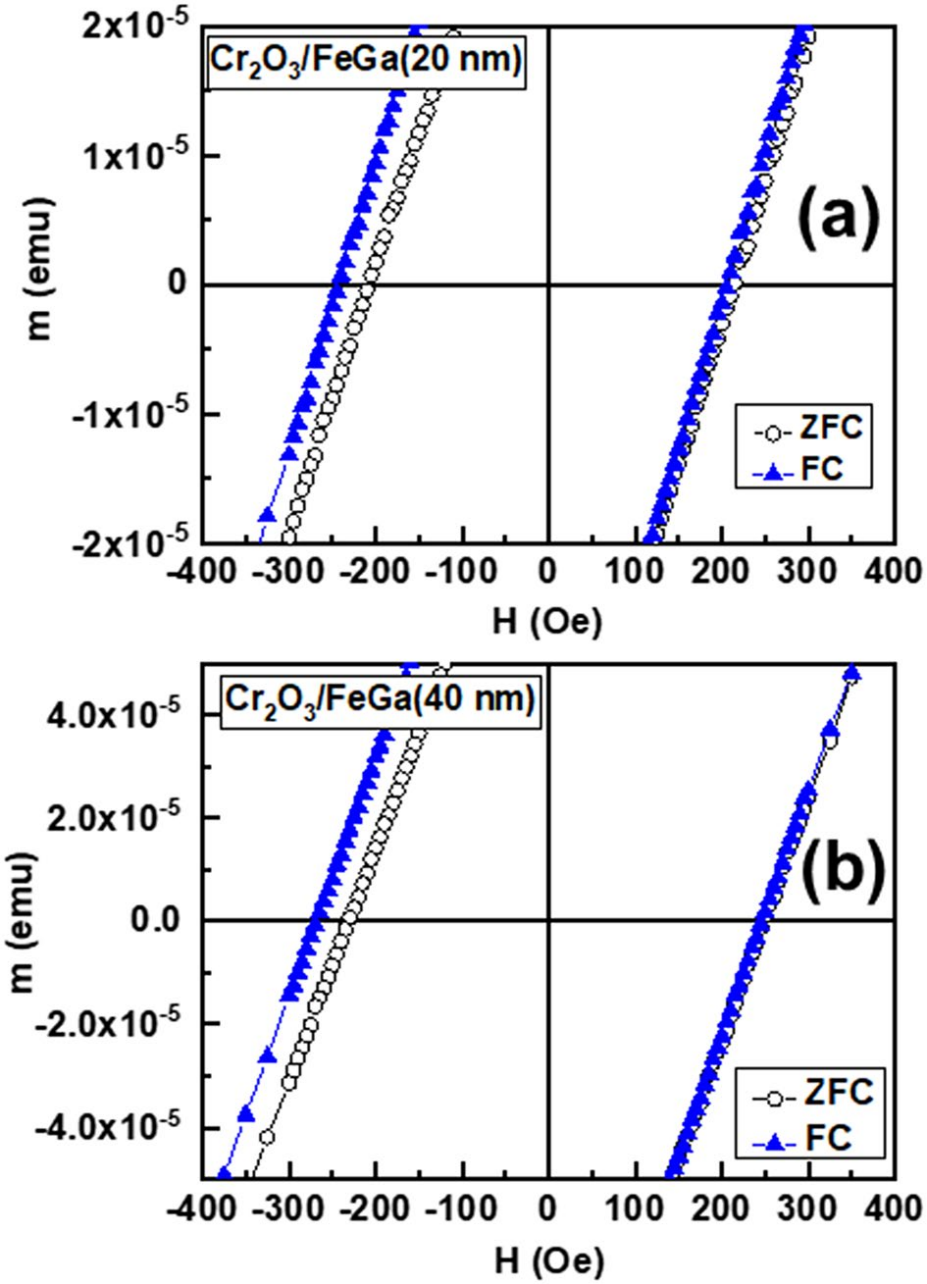
OOP hysteresis loops recorded at 5 K after FC at 1 kOe (filled triangle) and ZFC (open circle) from 360 K for samples with a FeGa thickness of (**a**) 20 nm, and (**b**) 40 nm.

In addition to the rotation of the Fe_72_Ga_28_ direction magnetization towards the perpendicular direction, we have observed an exchange-bias effect related to the Cr_2_O_3_/ Fe_72_Ga_28_ interfacial coupling as indicated by the shift of the OOP hysteresis loop in the horizontal axis (*H*_*E*_) at 5 K after a FC process at 1 kOe (Fig. 5). This exchange-bias phenomenon in the perpendicular direction is related to the exchange-coupling between the antiferromagnetic Cr_2_O_3_, and the ferromagnetic Fe_72_Ga_28_^41^. For a Fe_72_Ga_28_ thickness of 20 nm, *H*_*E*_ is − 21 Oe, and − 11 Oe for a thickness of 40 nm. For the lowest FeGa thickness, exchange-bias effect has been observed at least up to 200 K.

Since the perpendicular magnetic anisotropy inferred in this work for Fe_72_Ga_28_ (*K*_*FM*_ = 3·10^6^ erg·cm^−3^) is higher than the theoretical value of Cr_2_O_3_ (*K*_*AF*_ = 2·10^5^ erg·cm^−3^)^42^, and some chemical roughness is expected at the Fe_72_Ga_28_/Cr_2_O_3_ interface, we have used the random field model proposed by Malozemoff^35–37^ to calculate the theoretical *H*_*E*_ values. In this random field model, the AF layer breaks into domains, and the exchange-bias field is obtained by means of the expression:

6 $$H_{E} = \frac{{2z\sqrt {A_{{AF}} K_{{AF}} } }}{{\pi ^{2} M_{{FM}} t_{{FM}} }}~$$

where *z* is generally taken as the unity, and *A*_*AF*_ is the exchange stiffness of the antiferromagnet that takes a value of 4·10^−7^ erg cm^−1^^42^. With this expression (6) we obtain *H*_*E*_ equals to − 26 Oe and − 13 Oe for Fe_72_Ga_28_ thickness of 20 and 40 nm, respectively, that are pretty close to the experimental − 21 Oe and − 11 Oe, respectively. This well agreement confirms the possibility of using this model in Cr_2_O_3_-based exchange-biased systems as also previously reported^42^.

Finally, from *H*_*E*_ experimental values the interfacial exchange energy Δ*E* can be calculated:

7 $$\Delta E = H_{E} M_{{FM}} t_{{FM}}$$

For Fe_72_Ga_28_ of 20 nm and 40 nm, it is obtained a Δ*E* of 0.046 and 0.048 erg cm^−2^ that is similar than reported in previous works in which Cr_2_O_3_ has been coupled with other ferromagnets with values of 0.05 erg cm^−2^ at 5 K^30,41^. However, it is important to remark that we have obtained that interfacial energy using polycrystalline Cr_2_O_3_, not a fully *c*-oriented Cr_2_O_3_ with all the magnetic moments aligned in the perpendicular direction. Nevertheless, our experimental results point out that even in this situation, it is possible to gradually turn the Fe_72_Ga_28_ magnetization into the out of plane direction due to the combination of Cr_2_O_3_ with Fe_72_Ga_28_ that has a magnetic fluctuation that promotes magnetic ripple in the uncoupled layers^25^. Therefore, these results show the possibility of using polycrystalline layers for further applications such as tailoring of the magnetization direction.

## Conclusions

In summary, we have studied the effect of the interfacial coupling between an antiferromagnet with magnetic moments along the *c*-axis (Cr_2_O_3_) and a ferromagnet (Fe_72_Ga_28_) with the magnetization in the sample plane but with a fluctuation that produces magnetic ripple. First of all, the in-plane magnetic anisotropy of Fe_72_Ga_28_ is completely vanished due to the coupling. Secondly, stripe domains are promoted due to the enhancement of the OOP component of the Fe_72_Ga_28_ magnetization. We have observed that the magnetization direction of Fe_72_Ga_28_ is gradually turned from in-plane to the OOP direction as the Fe_72_Ga_28_ thickness is reduced A perpendicular magnetic anisotropy of 3·10^6^ erg·cm^−3^ has been inferred from experimental results in the Fe_72_Ga_28_ layers. It has also been observed exchange-bias phenomena in the perpendicular direction fitting the experimental data to the random field model.

## Supplementary Information

Supplementary Figure 1.
