# Supplementary material for: Interfacial coupling effect of Cr2O3 on the magnetic properties of Fe72Ga28 thin films
Source: Sci Rep. 2021 Jun 28;11:13429. doi: 10.1038/s41598-021-92640-y (PMC8239039; doi:10.1038/s41598-021-92640-y)
Supplement: Supplementary file 1 — Supplementary Figure 1. [file 41598_2021_92640_MOESM1_ESM.docx]

**Supplementary information**

**Figure S1.**

Power spectral density (PSD) calculated from MFM images for samples (a) Cr_2_O_3_/Fe_72_Ga_28_(40 nm) and (b) Cr_2_O_3_/Fe_72_Ga_28_(80 nm)


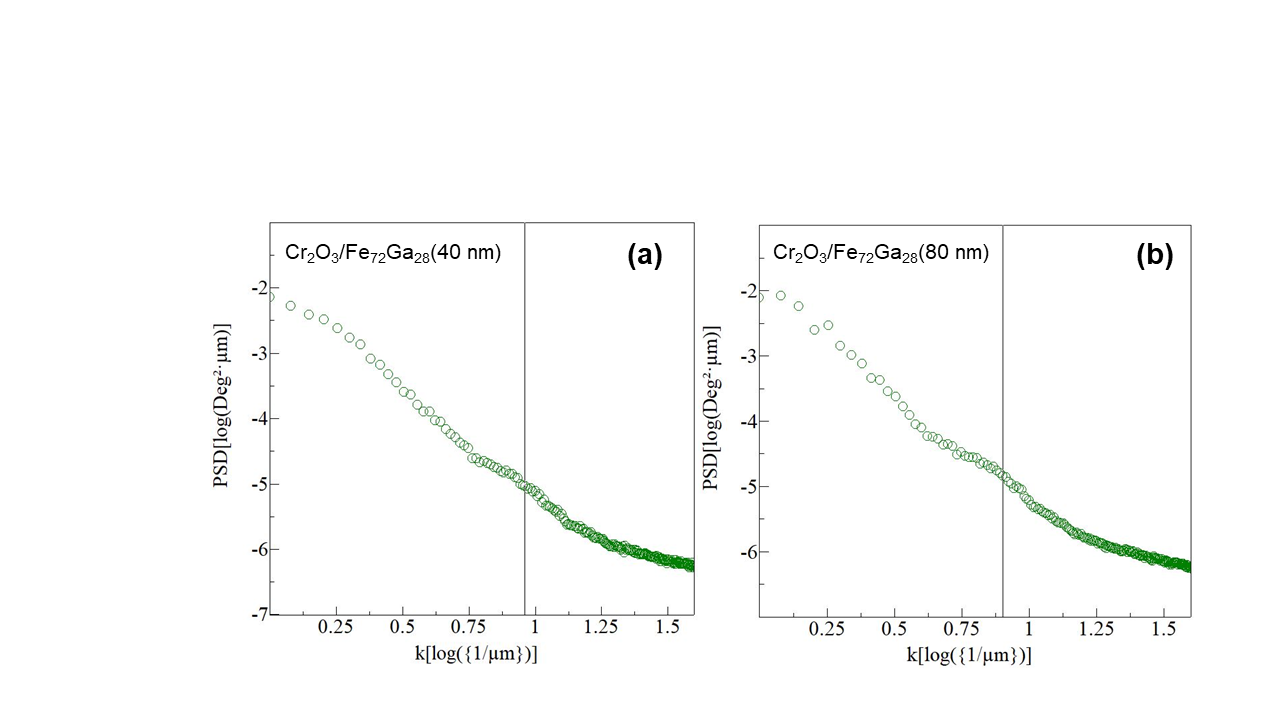


For the PSD we have used the program WSxM [I. Horcas *et al*. Rev. Sci. Instrum. **78**, 013705 (2007)].
